# Novel Tyrosine Kinase Targets in Urothelial Carcinoma

**DOI:** 10.3390/ijms22020747

**Published:** 2021-01-13

**Authors:** Javier Torres-Jiménez, Víctor Albarrán-Fernández, Javier Pozas, María San Román-Gil, Jorge Esteban-Villarrubia, Alfredo Carrato, Adriana Rosero, Enrique Grande, Teresa Alonso-Gordoa, Javier Molina-Cerrillo

**Affiliations:** 1 Medical Oncology Department, University Hospital Ramon y Cajal, 28034 Madrid, Spain; javier.torres.jim@gmail.com (J.T.-J.); vicalbarranfernandez@gmail.com (V.A.-F.); pozas.javier@gmail.com (J.P.); mariasanro21@gmail.com (M.S.R.-G.); jorge.e.villarrubia@gmail.com (J.E.-V.); 2 Medical Oncology Department, Ramón y Cajal Health Research Institute (IRYCIS), CIBERONC, Alcalá University, University Hospital Ramon y Cajal, 28034 Madrid, Spain; acarrato@telefonica.net; 3 Medical Oncology Department, Infanta Cristina Hospital, 28607 Madrid, Spain; adrirosero54@hotmail.com; 4 Department of Medical Oncology, MD Anderson Cancer Center, 28033 Madrid, Spain

**Keywords:** tyrosine kinase, tyrosine kinase inhibitors, urothelial carcinoma

## Abstract

Urothelial carcinoma represents one of the most prevalent types of cancer worldwide, and its incidence is expected to grow. Although the treatment of the advanced disease was based on chemotherapy for decades, the developments of different therapies, such as immune checkpoint inhibitors, antibody drug conjugates and tyrosine kinase inhibitors, are revolutionizing the therapeutic landscape of this tumor. This development coincides with the increasing knowledge of the pathogenesis and genetic alterations in urothelial carcinoma, from the non-muscle invasive setting to the metastatic one. The purpose of this article is to provide a comprehensive review of the different tyrosine kinase targets and their roles in the therapeutic scene of urothelial carcinoma.

## 1. Introduction

The urothelium is the epithelium that covers the surface of renal collecting tubules, calyces and pelvis in the kidney, and the surface of ureter, bladder and urethra. Urothelial or transitional cell carcinomas account for more than 90% of the cases. In terms of primary tumor origin, bladder cancer (BC) is the most frequent of the rest of the urinary system tumors. BC is the 10th most common type of cancer in both genders, with a total of 549,000 cases (3%) and 200,000 deaths (2.1%) in all around the world according to GLOBOCAN 2018. The incidence and mortality are, respectively, 9.6 and 3.2 per 100,000 in men, with an incidence and mortality almost four times lower in women. Incidence rates vary across the world, with a higher number of cases in Southern Europe, Western Europe and North America [1,2].

It is estimated that approximately 75% of new urothelial BC cases are classified as non-muscle-invasive bladder cancer (NMIBC). Muscle-invasive bladder cancer (MIBC) and metastatic disease (mUC) are less frequent, as new diagnoses. All these entities present important differences in pathogenesis, management and prognosis [3]. Therefore, this review will focus mainly on the advanced diseases, which are associated with 5-year survival rates of 60% and <10%, respectively. This data, along with a median time to progression (TTP) of 8–11 months to platinum-based therapy, is considered an urgent unmet need for the development of novel therapies that improve the dismal prognoses of these patients [4].

The heterogeneity and different outcomes identified in the subset of MIBC have been analyzed in order to establish an additional classification that enables the development of new therapeutic agents depending on the most common oncogenic pathways involved. An international consensus has examined and classified MIBC into six different subtypes with different characteristics and prognoses: basal-squamous (35%), luminal papillary (24%), luminal unstable (15%), stroma-rich (15%), luminal nonspecified (8%) and neuroendocrine-like (3%) (Table 1). The therapeutical implications could be even greater, since some of the subtypes, such as the basal-squamous one, present some similarities with other squamous cell carcinomas and may be placed in the same TCGA pan-cancer cluster, whose importance relies on the possible inclusion in basket clinical trials based on the molecular alterations over the histological type. However, there are no predictive biomarkers used in our daily routine, and further studies are required in this field [5].

The current therapeutic approach to mUC is represented by platinum-based chemotherapy (CT) and immunotherapy (IT) with PD-1/PD-L1 inhibitors in different scenarios [6,7]. Novel drugs with a different mechanisms of action have been recently approved for patients with locally advanced or mUC who have previously received immune checkpoint inhibitors (ICI) and/or platinum-based CT, such as erdafitinib, an FGFR inhibitor, and enfortumab vedotin, a monoclonal antibody-drug conjugate targeting nectin-4 [8,9]. Other chemotherapeutic treatments, such as taxanes, vinca alkaloids and pemetrexed may be useful as alternative regimens in selected patients according to their clinical situations [5,10,11].

The advances in the understanding the pathogenesis of urothelial carcinoma have led to the development of new strategies, such as targeted therapies and ICI after failure of platinum-based therapy. Next-generation sequencing (NGS) has enabled us to acquire better knowledge of the genetic characteristics of BC with a better characterization of potential therapeutic targets [12].

The aims of this review are to summarize the most important tyrosine kinase targets in mUC and the molecular characteristics that explain their effectiveness, and present the main ongoing clinical trials in this setting.

## 2. Targeting ERB-B: Epidermal Growth Factor Receptor (EGFR) and HER-2

### 2.1. Molecular Biology of the EGFR

The epidermal growth factor receptor (EGFR) is a member of the ErbB tyrosine-kinase receptors family, composed by EGFR (ErbB-1), HER2/c-neu (ErbB-2), HER3 (ErbB-3) and HER4 (ErbB-4). EGFR family proteins have key roles in the modulation of normal cell growth and differentiation but also well-established importance in malignant cell growth, proliferation and development of drug resistance.

Its main ligands are EGF, transforming growth factor alfa (TGF-alfa) and heparin-binding EGF-like growth factor. After being activated by ligand binding, it phosphorylates and recruits several downstream signaling molecules, and therefore activates several major pathways, including phosphatidylinositol-specific phospholipase C (PLC)-protein kinase C (PKC), Ras-Raf-MEK (MAPK), phosphatidylinositol 3-kinase (PI3K)/protein kinase B (Akt)/mammalian target of rapamycin (mTOR) and Janus kinase 2 (JAK2)-signal transducer and activator of transcription 3 (STAT3), which have been shown to participate in the regulation of cell growth and survival [13].

**Figure 1:**
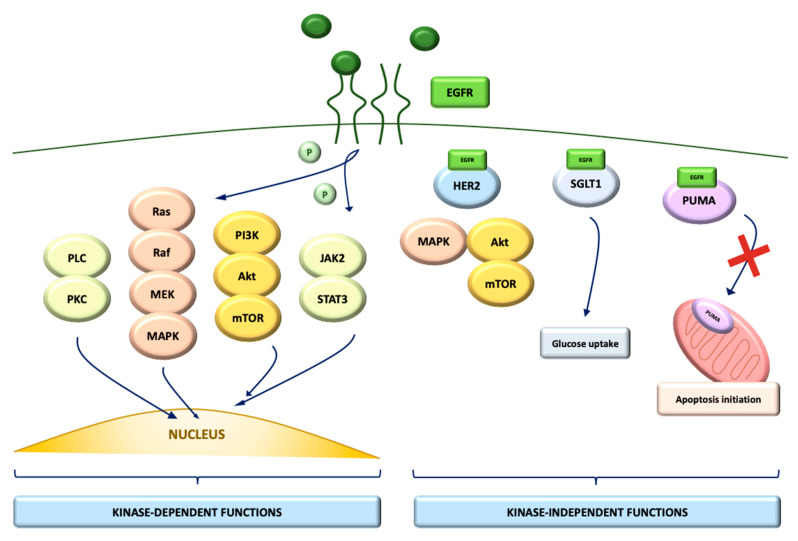
EGFR tyrosine-kinase dependent and independent signaling pathways.

However, besides its tyrosine-kinase dependent activity, EGFR can also mediate cellular processes through the capacity to physically interact with other proteins, such as sodium-glucose cotransporter 1 (SGLT1), and thereby modulate protein subcellular trafficking [14]. EGFR associates with p53-upregulated modulator of apoptosis (PUMA), a proapoptotic member of the Bcl-2 proteins family, and as a consequence of EGFR-PUMA interaction, PUMA is sequestered in the cytoplasm, avoiding it from being translocated onto the mitochondria to initiate apoptosis (Figure 1).

EGFR and PUMA seem to have a high co-expression in cell lines and primary specimens of different types of tumor cells, suggesting that EGFR overexpression might have anti-apoptotic and therefore oncogenic activity, even without the involvement of its kinase-dependent intracellular pathways [15]. According to these findings, targeting both kinase-dependent and independent functions of EGFR could be necessary for an effective strategy to overcome tumor resistance to conventional anti-EGFR therapies.

In addition to the above-mentioned cell-surface and cytoplasmic ways of EGFR signaling, EGFR can also be found in the nucleus and inside mitochondria of both normal and malignant cells.

Nuclear expression of EGFR has been detected in many different types of tumoral cells, including BC, localized within the nucleoplasm and on the inner nuclear membrane [16]. Nuclear EGFR seems to work as a transcriptional regulator of several genes, including those encoding cyclin D1, inducible nitric oxide synthase (iNOS), B-Myb, cyclooxygenase-2 (COX-2), c-Myc and breast cancer resistance protein (BCRP) [17,18,19]. An overexpression of EGFR may lead to enhanced proliferative and inflammatory activity in tumoral cells due to the increased activity of these target genes.

Besides working as a regulator of gene transcription, nuclear EGFR seems to retain its tyrosine-kinase activity, and it may contribute to phosphorylation of proliferating cell nuclear antigen (PCNA) to promote cell proliferation and DNA repair [20]. As nuclear EGFR seems to protect normal cells from DNA damage caused by ultraviolet and gamma irradiations, it also may play an essential role in DNA repair following radiation therapy, suggesting a potential benefit of combining radiotherapy with anti-HER2 therapy in patients with HER2-positive tumors [21].

Since EGFR-driven signaling is related to unregulated cell growth and division through several molecular pathways, EGFR gene mutations and amplifications have been considered targetable alterations in a variety of cancers. Recent studies have reported that nearly 14% of urothelial tumors have some amplification or overexpression of EGFR [22], these aberrations being associated with more aggressive forms of the disease and a tendency to developing CT resistance [23]. These findings have given rise to prospective analysis of several anti-EGFR targeted therapies in urothelial cancer, including cetuximab, panitumumab, gefitinib and lapatinib.

### 2.2. Molecular Biology of the HER2 Receptor

Ligand binding to EGFR (HER1), HER3 or HER4 leads to dimerization with HER2, forming heterodimers that are able to generate intracellular signals [24]. When HER2 is overexpressed, multiple HER2-containing heterodimers are formed and cell signaling is intensified, favoring oncogenesis as a result of a pathological reactivity to growth factor stimulation.

The HER2 receptor is encoded by the HER2 gene, a proto-oncogene mapped to chromosome 17q21, and it is composed of a cysteine-rich extracellular ligand binding site, a transmembrane segment and an intracellular domain with tyrosine-kinase activity [25]. According to current knowledge, no existing ligand binds directly to HER2, and heterodimer formation, induced by molecules known as neuregulins, follows EGFR, HER3 and HER4 interactions with ligands [26]. This has led some authors to propose that HER2 works as a ligand-less receptor for all HER ligands, including epidermal growth factor (EGF) and transforming growth factor alfa (TFG-alfa) [27]. HER2–HER3 heterodimers are the most mitogenic combination and HER3 seems to be the predominant partner of HER2 in malignant cells [28].

Negative regulation of HER2 receptor signaling is controlled by the endocytic removal of HER2 from the cell surface, activating a degradation pathway that seems to be controlled by c-Cbl, a tyrosine phosphorylation substrate of HER that also contains a ring finger domain which serves as a binding site for a ubiquitin processing enzyme (E2) [29]. Thereby, if c-Cbl is recruited to a phosphorylated residue of HER, it enhances the polyubiquitination and degradation of the receptor. This mechanism is of great relevance to cancer IT, since anti-HER2 monoclonal antibodies seem to recruit c-Cbl, enhance ubiquitination of HER2 and therefore accelerate its internalization and degradation, reducing its oncogenic effect [30].

Although anti-HER2 targeted therapy has been mainly developed in breast and gastric cancer, recent comprehensive molecular profiling has demonstrated an incidence of ErbB family mutations, amplifications and over-expression in up to 20–30% of BC patients [31], with particularly high rates of HER2 alterations in micropapillary bladder tumors [32]. Besides, HER2 expression seems to be lower in the primary tumor (28%) than in locoregional lymph node metastases (53%), suggesting that HER2 may have an impact on the systemic dissemination of BC [33].

Although HER2 has been undoubtedly proved a prognostic and predictive biomarker in human cancer, its clinical extrapolation is conditioned by the variability of reported HER2 alterations depending on the disease stage, the tested populations and both inter-tumor and intra-tumor heterogeneity. Besides, there seems to be a discordance between HER2 immunohistochemistry (IHC), fluorescence in situ hybridization (FISH) and genomic-level molecular characterization, since FISH rates have been generally lower than IHC rates for HER2 positivity in the published studies [34].

### 2.3. Clinical Trials of EGFR

Although the EGFR family was one of the first treatment targets in mUC, the goal has not been reached, with disappointing results in clinical trials. Identification of predictive biomarkers of EGFR therapy and pretreatment genomic characterization might improve the development of these drugs [23].

Several studies using gefinitib have been performed in combination with or after CT. A phase II study led by SWOG using gefitinib as single agent therapy was performed in 31 patients in whom conventional CT for mUC had previously failed. Almost half of the pretreatment biopsies expressed strong EGFR in IHC. Median Overall Survival (mOS) in patients in this study was 3 months and median Progression Free Survival (mPFS) was 2 months [35]. However, a phase II study using gefinitib combined with CT in naïve patients by CALGB (Cancer and Leukemia Group B) showed a mOS of 15.1 months and mPFS of 7.4 months. There was no improvement in the response rate or survival compared to those in a historical control with CT alone [36]. Miller et al. published a phase II study on 105 mUC patients. The results proved that adding gefitinib to CT did not improve the survival outcome [37].

Afatinib has also been studied in a phase II trial including 23 patients with platinum-refractory disease, reaching a 3-month PFS of 21.7% [38]. The median period to progression/discontinuation was 6.6 months in cases with HER2 or ERBB3 mutations compared with 1.4 months in wild type cases. These findings supported afatinib as a potential therapy in selected patients with HER2/ERBB3 alterations. A phase II is currently evaluating afatinib in mUC patients who have progressed to CT and harbor EGFR alterations (HER2/ERBB3 mutations, HER2 amplification, EGFR amplification) [39].

A randomized and non-comparative phase II study tried to measure the efficacy of cetuximab with or without paclitaxel in patients with previously treated mUC. The single-agent cetuximab arm closed early after nine of the first 11 patients progressed at week 8. The combination arm completed the full accrual of 28 patients. Overall response rate (ORR) was 25% (95% CI, 11% to 45%), mPFS was 16.4 weeks (95% CI, 12 to 25.1 weeks) and mOS was 42 weeks (95% CI, 30.4 to 78 weeks) [40]. Cetuximab has also been combined with cisplatin and gemcitabine in a randomized phase II trial. ORR was 57.1% for the CT arm and 61.4% for the combination arm. mPFS was 8.5 months for cisplatin–gemcitabine (95% CI = 5.7–10.4 months) and 7.6 months for cisplatin–gemcitabine–cetuximab (95% CI = 6.1–8.7 months). These results revealed that the addition of cetuximab was intolerable because of its high toxicity with no relevant improvement in survival outcomes [41].

Research in EGFR targets is ongoing, and there are promising EGFR target agents in preclinical phases. There is an EGF-conjugated anthrax toxin that after targeting EGFR is internalized and triggered apoptosis in BC cells. This EGF-toxin conjugate promoted its own uptake via receptor microclustering [42].

### 2.4. Clinical Trials of HER-2

Despite the fact that mUC has one of the highest rates of HER2 expression of any solid tumor (12.4% in urothelial carcinoma, 11.3% in esophagueal and esophagogastric junction cancers and 10.5% in breast cancer) [43], clinical trials analyzing HER2 targeting in those patients have not shown a clinically significant benefit.

A single arm phase II study evaluating trastuzumab in combination with paclitaxel, gemcitabine and carboplatin in 44 patients with HER2 positive mUC showed a 70% ORR and 14.1 months mOS [44]. Another phase II study compared CT with or without trastuzumab in HER2 positive mUC, but it was inconclusive because ORR, mPFS and mOS were similar between groups [45].

Lapatinib, a bifunctional EGFR and HER2 kinase inhibitor, has been evaluated as a second line therapy. A phase II study in 59 patients showed an ORR of greater than 10% in only 1.7% of patients, but 31% achieved stable disease. mPFS and mOS were 8.6 and 17.9 weeks, respectively. ORR correlated with EGFR overexpression [46]. However, a phase II study showed that the combination of lapatinib with CT was too toxic in patients with advanced UC [47]. Lapatinib has been evaluated as maintenance therapy in a phase II/III study in patients with EGFR and/or HER2 overexpressing locally advanced or mUC cancer (NCT00949455), but it did not show a clinical benefit of lapatinib compared with placebo [48]. Further research with new targets against HER2 is ongoing, such as trastuzumab–deruxtecan in combination with IT (nivolumab) in a multicohort phase I trial including patients with mUC (NCT 03523572).

## 3. Targeting Fibroblast Growth Factor Receptor (FGFR)

### 3.1. Molecular Biology of FGFR

Fibroblast growth factors (FGFs) are a family of 22 cell-signaling proteins of extracellular origin, generally released upon tissue injury, which act as systemic or locally circulating molecules capable of activating tyrosine-kinase receptors. They have been classified in seven subfamilies according to their phylogeny: five paracrine FGFs (FGF1, FGF4, FGF7, FGF9 and FGF8), an endocrine FGF (FGF15/19) and an intracellular subgroup (FGF11). These receptors have a beta-trefoil fold with a heparan sulfate binding-site that facilitates its sequestration close to the cell surface for binding to an FGF receptor (FGFR) [49].

FGFRs are encoded by four different genes (FGFR1–FGFR4) and are composed of three extracellular immunoglobulin-type domains (D1, D2 and D3), with D3 mediating heparan-sulfate binding and being primarily responsible for ligand specificity. The dimerization of the FGFR intracellular-domain precedes an autophosphorylation signal for the tyrosine-kinase domain that leads to the activation of several downstream transduction pathways [50].

**Figure 2:**
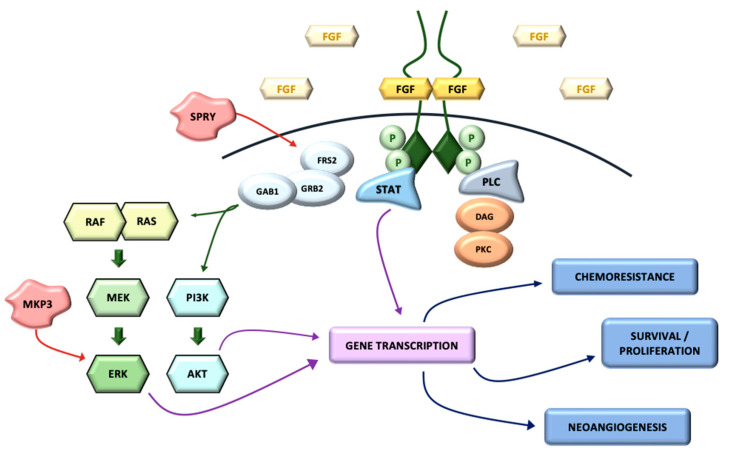
FGFR signaling pathway.

Mainly, two different mechanisms have been described in the further transmission of the signal. The first one is the activation of RAS-dependent mitogen activated protein-kinase (MAPK) and Raf phosphorylation. The second one leads to cell activation through other signaling molecules, such as Shb, Src kinase and STATs (signal transducers and activators of transcription), amongst others. The whole FGF/FGFR pathway is strongly regulated by feedback mechanisms, such us SPRY (which down-regulates the activation of growth factor receptor-bound protein) and MKP3 (which attenuates MAPK signaling) [51] (Figure 2).

In non-cancer cells, the activation of FGFRs leads to the stimulation of several intracellular signaling cascades that play crucial roles in embryonic development, metabolism and tissue repair. Due to the significant influences of FGF/FGFR pathway on cell growth, proliferation and differentiation, its dysregulation secondarily to different kinds of genetic aberrations (including receptor mutations, amplifications and chromosomal translocations) has an important oncogenic role, especially related to tumor progression and resistance to CT. Around 7.1% of all tumor types present genetic alterations in the FGF/FGFR axis, FGFR1 being the most frequently altered (49%), followed by FGFR3 and FGFR2—hence it is the third most frequently altered pathway after TP53 and KRAS [52].

Specifically, amplifications of *FGFR1* gene have been found in 9–10% of urothelial BC, followed by FGFR3 (3–5%) and FGFR2 (0.8%), and activating mutations of FGFR3 gene have been described in 38–66% of non-invasive BC and 15–20% of invasive BC. Interestingly, for therapeutic purposes, the presence of any FGFR mutation, fusion or overexpression seems to be associated with a higher sensitivity to FGFR inhibitors in pre-clinical models [53].

Amplification of FGFR represents around 66% of FGFR alterations, with FGFR1 being the most frequently amplified subtype. FGFR1 amplification seems to be much more represented in early than advanced-stage tumors, suggesting a possible role of FGFR1 amplification during the initial phase of oncogenesis, which may be clinically relevant for therapeutic purposes [54].

Missense mutations such as *FGFR3^S249C^* (21%), *FGFR3^Y375C^* (7%), *FGFR3^R248C^* (3%) and *FGFR3-TACC3* fusions (2%) are relatively common in NMIBC (20–50%) and not rare in MIBC (10%) [55], and they have been related to the aberrant formation of cys-mediated intermolecular bonds between mutant receptors and to the constitutive activation of the FGFR3 tyrosine-kinase [56,57].

Despite these genetic alterations having set the stage for the development of targeted therapies, the modest response rates observed in clinical trials, and the accumulating evidence related to other TKIs, suggest that primary or acquired resistance is an unavoidable concern related to the current FGFR inhibitors. The bypass activation of the same or similar downstream effectors is a known mechanism of both intrinsic and acquired resistance. For example, the activation of EGFR/HER3-dependent PI3K/Akt signaling has been described in urothelial tumors harboring driver FGFR3 mutations such as *FGFR3^S249C^* and *FGFR3-TACC3*, which are intrinsically resistant to FGFR3 inhibition, suggesting that EGFR-dependent PI3K signaling is a potential mechanism of resistance to FGFR inhibitors [58]. A second major cause of resistance to FGFR-targeted therapies is the emergence of secondary *FGFR* alterations. Gatekeeper mutations, including *FGFR1^V561M^*, *FGFR2^V564F/I^*, *FGFR3^V555M^* and *FGFR4^V550E/L^*, can either occur de novo or during treatment with targeted therapies, leading to amino acid substitutions for the valine residue located in the drug-binding pocket of the tyrosine-kinase domain that may alter the mode of drug-FGFR interactions [59]. Intratumor heterogeneity has been also considered involved in the antitumor responses to FGFR targeted therapies. The homogeneous overexpression of FGFR has been shown to confer malignant cells a high sensitivity to FGFR inhibitors, whereas a heterogeneous FGFR upregulation might entail the existence of resistant cell clones.

Further research is necessary to adequately monitor and identify the emergence of resistant tumor subclones with an activation of parallel pathways or secondary *FGFR* mutations, enabling the detection of treatment resistance and the stratification of patients to receive appropriate targeted therapies.

### 3.2. Clinical Trials in FGFR

Several compounds have been developed in recent years to inhibit FGFR. Some of them are non-selective multi-target inhibitors, and others are highly selective FGFR-TKIs, although other approaches, such as monoclonal antibodies and FGF-ligand traps, are also under research. Table 2 shows the more relevant clinical trials targeting FGFR.

Erdafitinib is a novel pan-FGFR kinase inhibitor recently approved by the FDA for patients with locally advanced cancer or mUC with susceptible FGFR3 or FGFR2 genetic alterations who have progressed during or following platinum-based CT [60]. Approval was based on data from the primary analysis of the BLC2001 study [61]. The final results of this phase II trial were presented at ASCO 2020, including long-term outcomes and safety data. With a median follow-up of 24 months, the investigators confirmed an ORR of 40%, with a median duration of response of 6 months. Furthermore, 31% of responders had a duration of response over 12 months. mPFS was 5.52 months and mOS was 11.3 months. Central serous retinopathy (CSR) occurred in 27% (27/101) of patients, but 85% of those (23/27) were grade 1 or 2 [62]. In addition, a phase III trial is evaluating erdafitinib compared to pembrolizumab or CT in patients with mUC and FGFR alterations who have progressed after one or two prior treatments (NCT03390504) [63].

Furthermore, the combination of FGFR inhibition and IT has been analyzed with different agents. The rationale for this strategy is based on different hypothesis. IT may enhance the antitumor effects of FGFR inhibitors and also prevent or delay the development of resistance. Urothelial carcinoma can be divided into T-cell-inflamed and non-T-cell-inflamed subtypes [64]. Non-T-cell-inflamed subtypes correlated with an absence of CD8+ T lymphocyte and resistance to IT, which produced a rationale for a combination of FGFR inhibitors and anti-PD-1/PD-L1 [65]. The aim of the combination of an FGFR inhibitor and an anti-PD-1/PD-L1, such as NORSE study, FORT-2 or FIGHT-205, is to prove that targeting FGFR makes it possible to turn an immunologically cold tumor into a hot tumor.

Therefore, a phase Ib/II clinical trial (NORSE study) evaluated erdafitinib in combination with cetrelimab, a PD-1 inhibitor, in 15 patients with mUC and FGFR2/3 alterations after progression to at least one line of treatment. The combination of erdafitinib (8 mg with uptitration to 9 mg) with cetrelimab was deemed safe for further evaluation [66]. In the seven patients treated with the recommended phase II dose, ORR was 71%. This combination is further being evaluated in a randomized phase II clinical trial in combination with platin-based CT (NCT03473743). However, in high risk, BCG refractory NMIBC with FGFR gene alterations, erdafitinib is being compared with intravesical CT (NCT 04172675).

Infigratinib (BGJ398) is an oral, selective, ATP-competitive FGFR 1–3 TKI. A phase I clinical trial evaluated the safety and antitumor activity of infigratinib in 132 patients with solid tumors [67]. Thirty-three mUC patients with activating FGFR3 mutations or fusions received BGJ398 125 mg on a once-a-day, 3 weeks on/1 week off regimen. Median treatment duration was 13.3 weeks. ORR was 35% [68]. This drug is under development in other UC settings, such as in the perioperative context and in upper urothelial tract (a promising response has been identified in a phase I trial [69]). A phase III clinical trial is currently evaluating infigratinib in patients with UC in the bladder and upper tract in the adjuvant setting (NCT04197986) [70].

Rogaratinib is an oral and selective FGFRs 1–4 TKI that inhibits the auto-phosphorylation of FGFR. A phase I trial tested rogaritinib in patients with advanced solid tumors who were FGFR mRNA-positive. In the mUC cohort, the ORR was 20.8%, with one patient achieving a complete response, and the disease control rate (DCR) was 68.1% [71].

The FORT-1 study evaluated the efficacy of rogaratinib in comparison with CT in patients with mUC who received prior platin-based CT [72]. Patients were included according to FGFR 1 and 3 mRNA expression, analyzed centrally by in situ hybridization from archival tumor tissue; 175 patients were randomized in the study—87 to the rogaratinib arm and 88 to the chemotherapy arm. The ORRs were 19.5% and 19.3% (1-sided *p* = 0.56), and mPFS values were 2.7 (95% CI, 1.6–4.2) vs. 2.9 (95% CI, 2.6–4.2) months for rogaratinib and CT, respectively. In the exploratory analysis directed at patients with FGFR3 DNA mutations or fusions, ORR was 52.4% for rogaratinib—higher compared to CT’s 26.7%. Considering these results, the study terminated early.

FORT-2 is a phase Ib/II study that evaluates the safety and efficacy of rogaratinib in combination with atezolizumab, an anti PD-L1, as a first-line treatment in cisplatin–ineligible patients with mUC and FGFR mRNA overexpression. The ORR was 44%, with a DCR of 68% and the duration of response was not reached. The most common treatment-emergent events were diarrhea (58%), hyperphosphatemia (45%) and urinary tract infection (36%). The presence of resistance gene mutations was analyzed, and three patients with detectable mutations in PI3K had no objective response [73].

Pemigatinib is another potent and competitive oral inhibitor of the kinase activity of FGFRs 1, 2 and 3. There was a phase II clinical trial (FIGHT-201) with mUC patients who progressed on one or several lines of therapy or were platinum ineligible [74]. Sixty-four patients with some FGFR3 mutation or fusion were assigned to cohort A, and 36 patients with other FGF/FGFR genetic mutations were assigned to cohort B and received pemigatinib. ORR was 25% (95% CI, 14–40%). The efficacy of pemigatinib in combination with pembrolizumab was compared with the standard of care (CT or IT) in patients with cisplatin-ineligible UC in a phase II randomized study (FIGHT-205, NCT04003610).

TAS-120 is a selective irreversible inhibitor for FGFR 1–4. A phase I study treated 134 patients with different advanced solid tumors and FGFR aberrations. Twenty-one mUC patients were included. In the dose-escalation phase, a 20 mg per day oral dose of TAS-120 was considered safe and exhibited clinical activity in various tumors, which need to be confirmed [75]. 

Debio-1347 is a small oral molecule that selectively inhibits the ATP binding site of FGFR1–3. A phase I clinical trial evaluated the safety and antitumor activity of debio-1347 in 58 patients with solid tumors with FGFR 1–3 alterations; 10% of patients had mUC [76]. 

Dovitinib is a small multikinase inhibitor that binds to FGFR3, inhibiting its phosphorylation. A phase II trial was prematurely closed because the ORR was 0% in FGFR3-mutated and FGFR3 wild-type patients [77]. Dovitinib in patients with localized UC did not show a clinical benefit in a phase II trial [78].

Derazantinib is a potent ATP competitive multikinase inhibitor of FGFR 1–3 and the colony stimulating factor 1 receptor (CSF1R) kinase. FIDES-02 is a clinical trial that is evaluating the safety and antitumor activity of single-agent derazantinib or in combination with atezolizumab in patients with mUC and FGFR aberrations (NCT04045613).

Recently, the BISCAY study (NCT02546661), characterized as an ambitious study on prospectively adapting the treatment based on genetic alterations, did not achieve a significant benefit for the patients included. Thus, in patients with FGFR, homologous repair gene or mTOR alterations, the study failed to significantly improve the ORR of 27.6% with durvalumab alone compared to AZD4547+durvalumab (ORR = 28.6%), olaparib+durvalumab (ORR = 35.7%) or vistusertib+durvalumab (ORR = 24.1%) [79].

In general, FGFR inhibitors share some adverse events (AEs) which are most easily manageable, but that require close physical examination monitoring, ophthalmic evaluation and early supportive therapy when required (Table 3) [80].

## 4. Targeting the PI3K/AKT/mTOR Pathway

### 4.1. Molecular Biology of the PI3K/AKT/mTOR Pathway

The PI3K/Akt/mTOR signaling pathways have important roles in regulating many aspects of cell growth and survival, being heavily interconnected with many other pathways; that is why these paths have a crucial role in oncogenesis and tumor biology [81].

PI3K is a family of lipid kinases with the capacity to phosphorylate inositol phospholipids, further divided into two subclasses: IA (activated by tyrosine-kinase receptors) and IB (activated by receptors coupled with G proteins). PI3K stimulation leads to the recruitment of signaling proteins such as Akt/PKB, and the production of second messengers that regulate several processes involved in cell cycle modulation [82]. The activation of the PI3K pathway is related to the MAP-K pathway (Raf/MEK/ERK), since both of them highly depend on KRAS stimulation.

**Figure 3:**
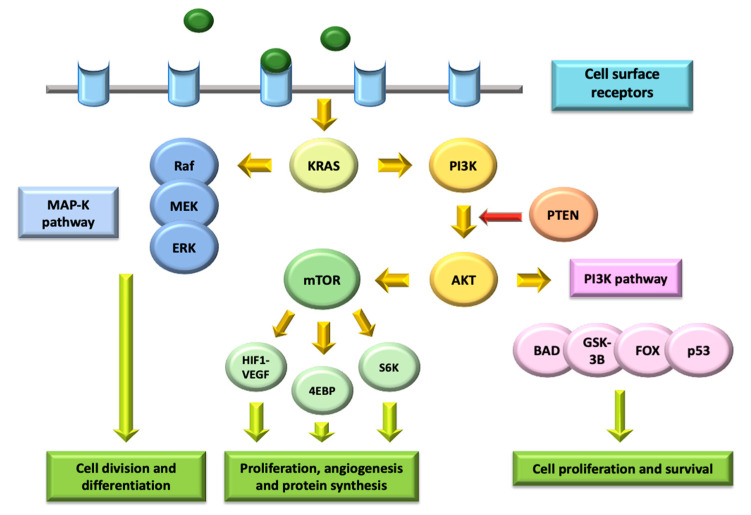
Phosphatidylinositol-3-kinase (PI3K)/Akt/ mammalian target of rapamycin (mTOR) pathways and their interaction with mitogen-activated protein-kinase signaling.

Akt is part of AGC kinase family, and its structure includes three conserved domains (N-terminal, central kinase CAT domain and C-terminal extension). It is able to inactivate proapoptotic factors such as BAD and procaspase-9, hence inhibiting programmed cell death and promoting proliferation, and it can also activate positive regulators of survival factor NFkB, such as IkB kinase [83]. Besides, several targets of Akt are involved in protein synthesis, glycogen metabolism and cell cycle regulation, especially regarding a positive modulation of G1/S progression through the inactivation of GSK3 [84] (Figure 3).

Negative regulation of PI3K/Akt pathway is mainly carried out by PTEN, a PI3,4,5-P3 phosphatase with the capacity to inhibit cell growth and enhance cellular sensitivity to apoptosis, thereby acting as a negative modulator of PI3K signaling and presenting tumor suppressor activity. In fact, since the loss of PTEN leads to permanent PI3K/Akt activation, its mutations in germ-cell lines result in a higher risk of different malignancies, including urothelial cancers [85].

Mammalian target of rapamycin (mTOR) is a molecular complex whose activation leads to increased synthesis of a wide range of essential proteins. It contains two key components: mTOR-C1 (made up of mTOR, RAPTOR, mLST8 and PRAS40) and mTOR-C2 (composed of mTOR, RICTOR, Sin1 and mLST8). With a higher sensitivity to rapamycin, and therefore being the target of first-generation mTOR inhibitors, mTOR-C1 complex is able to activate S6K and 4EBP1, promoting translation and cell growth [86]. Meanwhile, mTOR-C2 complex promotes cell survival and proliferation through interacting with PI3K/Akt and Ras/MEK/ERK pathways [87].

mTOR1 contains the regulatory-associated protein of mTORC1 (RAPTOR) and mTORC2 contains the rapamycin-insensitive companion of mTOR (RICTOR) and mSIN1, which negatively regulates mTORC2 [88].

The mTORC1 pathway is regulated by PI3K/Akt and Ras-MAPK. mTORC1 activation by Akt requires the activation of mTORC2 activation, phosphorylation of Akt by mTORC2 (and by PDK1) and the phosphorylation of TSC2 (tuberous sclerosis complex-2) by activated Akt, which inhibits the TSC1 (tuberous sclerosis complex-1) and TSC2 combination. The activator of mTORC1, RHEB, is constitutively down-regulated by TSC1/2. The inhibition of TSC1/2 leads to the release of RHEB and mTORC1 activation in lysosomes [89,90].

As one of the most studied and well-described pathways in tumor oncogenesis, PI3K/Akt/mTOR appears to play a crucial role in urothelial cancer cell growth and survival. Overactivation of the PI3K signaling pathway in muscle-invasive and metastatic BC has been demonstrated in multiple independent studies. Mutations in PI3KCA are present in 21–25% of patients [91] and loss of PTEN expression can be found in 39–94% of cases [92]. A small proportion of the patients have tumors with less common aberrations, such as inhibiting mutations in PTEN (3–4%) and activating mutations in AKT1 (2–3%) [93].

The PI3K/Akt signaling pathway can be targeted by different categories of compounds that inhibit PI3K, AKT, mTORC1 or mTORC2. Since the first generation of rapamycin analogs and mTOR inhibitors, both selective and multi-target PI3K/Akt inhibitors have been developed and may have a crucial role in advanced BC treatment in the forthcoming years.

### 4.2. Clinical Trials in PI3K/AKT/mTOR Pathway

Everolimus is a small molecule that inhibits mTOR. Seront et al. [94] and Milowsky et al. [95] led two distinctive phase II trials with everolimus as a second-line treatment in 37 patients and 45 patients, respectively, with mUC. They noticed that given as a single agent, everolimus demonstrated a negligible RR. Milowsky reported that grade 3/4 toxicities were fatigue, infection, anemia, lymphopenia, hyperglycemia and hypophosphatemia. A phase II trial showed that everolimus was ineffective as a second line treatment after CT. Anemia (28%), peripheral neuropathy (28%) and fatigue (24%) were the most frequent grade 3–4 AEs [96]. However, Iyer et al. conducted a trial with everolimus, and one patient with an inactivating TSC-1 mutation had significant shrinkage and a durable response [97].

BEZ235 is an oral pan-class I small molecule that inhibits PI3K and mTOR. A phase II trial evaluated BEZ235 as a second line treatment in 20 mUC patients after CT treatment. One partial response and two stable diseases were reported in patients who did not have any PI3K/AKT/mTOR mutations. BEZ235 showed poor clinical activity, with a minority of patients showing clinical benefits [98].

Buparlisib (BKM120) is a pan isoform small molecule that selectively inhibits PI3K. A phase II trial in 13 metastatic mUC, previously treated with CT, described an mPFS of 2.77 months (95% CI: 1.83–3.71). Six patients displayed stable disease (one of which presented with a TSC1 mutation), and there was one partial response in a patient with a TSC1 mutation [99].

## 5. Targeting Angiogenesis

### 5.1. Molecular Biology of Angiogenesis

Angiogenesis has been defined as a hallmark of malignant cells, since it is crucial for adequate nutrient and oxygen supplies and is directly involved in the promotion of metastasis. The intravasation of tumoral cells facilitates the release of paracrine factors within the tumor environment that are able to activate KF-kB and ERK signaling pathways through binding to toll-like receptors [100].

**Figure 4:**
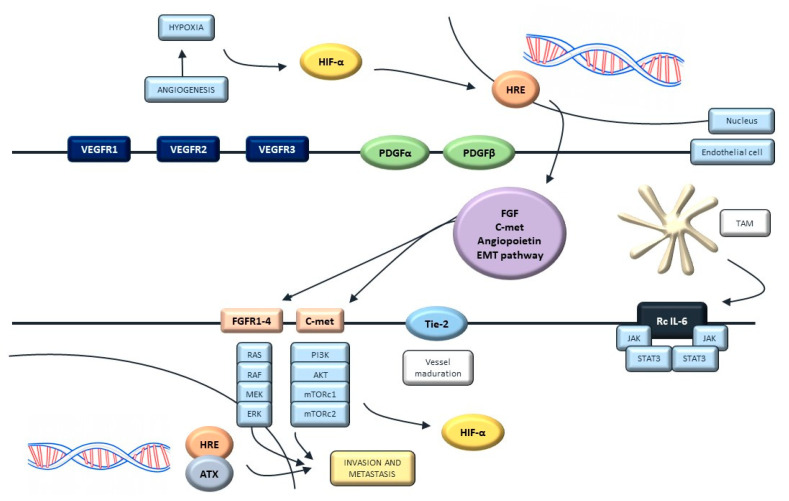
Angiogenesis pathways and their interaction with the MAP-K pathway.

Among these paracrine molecules, the vascular endothelial growth factor (VEGF) has been proved as a master regulator of tumor angiogenesis, promoting increased vascular permeability [101]. The hypoxia-inducible factor 1 (HIF-1) regulates VEGF expression. Oncogenic mutations affecting the RAS pathway and receptor crosstalk with other growth factors can stimulate VEGF expression [102]. The binding between VEGF and its receptor (VEGFR1 and VEGFR2) induces cell proliferation via the MAP-k pathway (RAS/RAF/ERK) and increases vascular permeability through the activation of SRC signaling (Figure 4) [103].

Overexpression of HIF-1 and VEGF has been demonstrated in urothelial tumors samples, and has been associated with a worse prognosis and higher rates of disease progression, recurrence and metastatic dissemination [104]. Recent studies have defined a high expression of VEGFA (main ligand of VEGFR) as an independent factor for poor prognosis in clinical cohorts of patients with advanced urothelial cancer [105]. Additionally, VEGFR serum levels over 400 ng/mL seem to be as a surrogate marker for metastatic disease identification [106]. In addition, vessel density is a predictor of vascular invasion, recurrence and shorter times of survival in invasive UC [107].

### 5.2. Clinical Trials Targeting Angiogenesis

Monoclonal antibodies and VEGF traps have reached phase III trials to analyze the role of angiogenesis inhibition in mUC. However, results have not led the approval of those drugs in this setting. Research is now focused on TKI, such as cabozantinib and lenvatinib. 

Bevacizumab is a VEGF monoclonal antibody. Different phase II studies have analyzed the combination of bevacizumab and CT as a first-line treatment in mUC patients. The ORR achieved was 72% and mOS was between 14 and 19 months [108,109]. Gemcitabine and cisplatin with bevacizumab or placebo was tested as a first line treatment in a phase III trial including 506 mUC patients. The study did not achieve the primary endpoint with a similar survival outcome in both arms [73]. There are two ongoing phase II trials investigating bevacizumab with atezolizumab in cisplatin-ineligible and previously untreated patients (NCT03133390, NCT03272217). Ramucirumab is a fully human monoclonal antibody designed to bind to a VEGFR-2 epitope involved in ligand binding, so it prevents VEGF ligands from binding this site. Though the promising results from the phase II trial in combination with docetaxel, this combination did not achieve clinically significant activity in the phase III trial [73,110]. Aflibercept is a recombinant fusion protein that binds and neutralizes multiple VEGF isoforms. In a phase II trial, aflibercept was administered as a single-agent in 22 mUC previously treated with chemotherapy and aflibercept showed grade 3 toxicities: fatigue, hypertension, proteinuria, pulmonary hemorrhage, pain, hyponatremia, anorexia and lymphopenia [111].

Sunitinib and pazopanib are small molecules that target VEGFR-1, VEGFR-2, VEGFR-3, c-KIT, PDGF-α and PDGFR-β. Sunitinib has been analyzed in different treatment scenarios and as monotherapy or in combination with CT. However, although some activity was identified, treatment toxicities were the major concern [112,113,114,115,116]. Pazopanib has also showed limited activity in previously treated patients as monotherapy or in combination with vinflunine or paclitaxel, with safety concerns [117,118,119,120,121].

Cabozantinib is a potent oral inhibitor of the tyrosine kinases c-Met, VEGFR2 and AXL. [122,123,124] A phase II trial with 41 relapsed mUC patients previously treated with platinum-based therapy showed a mOS of 8.2 months (95% CI: 5.2–10.3) and ORR of 19.5% (95% CI: 8.8–34.9) [122]. An open-label, single-arm, three-cohort phase 2 trial enrolled 68 patients previously treated with CT. ORR was 19% (95% CI: 9–34). The most common grade 3–4 AEs were fatigue (9%), hypertension (7%), proteinuria (6%) and hypophosphatemia (6%) [123]. It has been suggested that cabozantinib has immunomodulatory effects by decreasing the number of myeloid-derived suppressor cells and regulatory T cells, and increasing PD-1 expression in regulatory T-cells and the ratio of effector lymphocytes TCD8+over regulatory T cells [124]. Therefore, the combination of cabozantinib with ICI is now under investigation. In the expansion cohorts from a phase I trial (NCT02496208), the antitumor activity from cabozantinib plus nivolumab or nivolumab and ipilimumab showed promising results with an ORR of 38.5% (95% CI, 13.9–68.4%); the median duration of response was not reached; and the mPFS and mOS were 12.8 months (95% CI 1.8–24.1) and 25.4 months (95% CI 6.9–18.8), respectively [123]. Different phase II and III trials are ongoing, exploring the combination of cabozantinib plus ICI in mUC, such as atezolizumab in the COSMIC 021 trial (NCT03170960), among others.

Lenvatinib has also provided promising results by its antiangiogenic activity over VEGFR, FGFR, PDGFR and kit. Similarly to cabozantinib, lenvatinib may be immunomodulatory effects by decreasing myeloid-derived suppressor cells and regulatory T cells. Both cells possess immune-tolerant effects, and stimulate angiogenesis, TGF-beta secretion and non-inflammatory interleukins. Drugs such as cabozantinb and lenvatinib could revert this microenvironment to an immune-stimulant one [125,126]. Initial antitumor activity from the combination of lenvatinib and pembrolizumab (PD-1 inhibitor) was analyzed in a phase Ib/II trial that included 137 patients harboring different tumor types, including 20 patients with mUC (NCT02501096). The ORR at week 24 in this cohort was 25% (5/20; 95% CI, 8.7–49.1%). The most common treatment-related AEs were fatigue (58%), diarrhea (52%), hypertension (47%) and hypothyroidism (42%) [127].

## 6. Other Targets in Urothelial Carcinoma

### 6.1. Targeting the Anaplastic Lymphoma Kinase (ALK)

Anaplastic lymphoma kinase (ALK), so called after its discovery in anaplastic large-cell lymphoma cells, is a transmembrane protein that plays a crucial role in the embryogenesis and normal function of nervous system, since it regulates neuromuscular junction, retinal axon targeting and synapse development [128].

It has an extracellular ligand-binding domain that is strongly activated by two small secreted peptides (FAM150A and FAM150B), leading to a dimerization reaction and a change of structural conformation that activates its kinase domain, thereby phosphorylating residues that work as binding sites for the recruitment of other cellular proteins (GRB2, FRS2, PLC, PI3K, NFI, IRS1, Shc, Src, PTPN11) and therefore stimulating multiple downstream signaling mechanisms, including MAP-k, PI3K/AKT and JAK-STAT pathways [129].

Although translocation is the most frequent alteration of the ALK gene, and it has been mainly studied in non-small cell lung cancer, other genetic anomalies have also been described as pro-oncogenic events in different kinds of tumors. Preclinical studies have shown that malignant cells with ALK aberrations nearly completely depend on ALK intracellular signaling mechanisms for survival, explaining why their proliferation can be stopped by the inhibitory activity of specific targeted drugs [130].

This has led to the concept of beyond-organ “ALKomas” [131], meaning that due to the increasing evidence that ALK alterations are seen in malignancies from different origins, they should be stratified according to their oncogenic genotypes instead of their tissue types when considering therapeutic strategies. Bellmunt J. et al. found that ALK gene alterations, defined as minor copy number alterations (CNA) in the proximity of ALK locus detected by array comparative genomic hybridization (aCGH) were only present in 3 out of 96 (3.1%) tissue samples from patients with advanced urothelial cancer [124]. This may suggest a very low prevalence of ALK-activating mutations in advanced BC, entailing that a significant therapeutic benefit from ALK inhibitors in BC might be restricted to a select group of patients [132].

A single-arm, two-stage phase II study with crizotinib is evaluating its effectiveness in patients with c-MET or RON (receptor originated from Nantes)-positive mUC (LCI-GU-URO-CRI-001, NCT02612194). Additionally, a phase IIa study is currently evaluating trastuzumab/pertuzumab, erlotinib, vemurafenib/cobimenitib, vismodegib, alectinib (ALK) and atezolizumab in patients with advanced solid tumors and mutations or gene expression abnormalities predictive of response to one of these agents. This study started on April 2014 and the date of primary completion date is set as March 2021 (NCT02091141).

### 6.2. Targeting NOTCH Pathway

NOTCH is a transmembrane protein that has been involved in the maintenance of stem and precursor cells of normal tissues [133]. Its signaling route is activated by the interaction between the receptor and several ligands (jagged canonical NOTCH ligand, JAG, delta-like canonical NOTCH ligand, DLL) inducing the release of its intracellular domain (NICD) by two proteolytic steps, including cleavage by a gamma-secretase [134]. Once NICD has translocated to the nucleus, it binds to the CSL complex and recruits coactivators that trigger the transcription of several genes related to cell cycle modulation (HES1, HEY1) [135].

In malignant cells, NOTCH pathway seems to have both oncogenic behavior and tumor-suppressive activity depending on the tissue and tumoral microenvironment, since factors such as chromatin accessibility, noncoding RNAs and crosstalk with other pathways also play roles in the modulation of the genes transcriptionally regulated by NOTCH [136]. Regarding its oncogenic activity, several translocations and point mutations can induce the hyperactivation of NOTCH pathway and therefore lead to cell differentiation blockade and anti-apoptotic activity, facilitating cell proliferation and oncogenesis [137].

According to the studies that have demonstrated the involvement of NOTCH signaling in BC, NOTCH1 and NOTCH2 seem to have different roles that may be harnessed for therapeutic benefit. NOTCH1 expression is decreased in BC and its activation in cell lines reduces cellular proliferation, suggesting a tumor-suppressive role [138], whereas NOTCH2 seems to promote cellular proliferation and metastasis, therefore acting as an oncogene [139].

Some preclinical studies have reported that an individual NOTCH receptor may play opposite roles in the same tumor [140]. The differences in downstream signaling that may lead NOTCH pathway to play an oncogenic versus tumor-suppressive activity are still poorly studied, and molecular context may be critical for properly understanding NOTCH aberrations. Apart from this ambivalent oncogenic/tumor-suppressor activity, recent studies have shown NOTCH signaling is also involved in positive regulation of angiogenesis. Specifically, JAG/NOTCH interaction seems to suppress VEGFR/Sflt-1 and promote endothelial cell interactions, and in fact some experimental agents that suppress JAG-mediated NOTCH1 signaling have shown to inhibit angiogenesis and tumor growth [141]. NOTCH regulation by micro-RNAs (miRNAs), whose role in cancer biology has only begun to be understood, also seems to be implicated in a wide range of cellular processes, including cell proliferation and angiogenesis [142].

Given this rational, multiple molecular therapies have emerged during the last few years targeting the NOTCH pathway through different mechanisms, including inhibitors of gamma-secretase and DLL ligand; NOTCH receptor-targeted antibodies; and inhibitors of the NOTCH transcription complex.

### 6.3. Targeting c-MET and SRC

Tyrosine-kinase c-Met is a protein encoded by proto-oncogene MET, and it works as a receptor for the hepatocyte growth factor (HGF). Their interaction triggers the activation of crucial intracellular molecular pathways, such as PI3K/AKT, Ras/MAPK and JAK/STAT [143], leading to the regulation of several cellular processes, including angiogenesis, cell proliferation and differentiation.

Several studies have shown that c-Met overexpression may interfere with AKT/GSK signaling in BC, promoting cell migration and invasion [144]. In addition, c-Met phosphorylation participates in the activation of several intracellular pathways that have been involved in the promotion of epithelial–mesenchymal transition, invasiveness and drug resistance of urothelial malignant cells—the high expression of phosphorylated c-Met being a poor prognosis factor in patients with advanced BC [145].

Although properly understanding the involvement of c-Met in the progression of BC requires further research, several studies have shown that c-Met overexpression occurs in localized tumors, suggesting that targeted therapies against c-Met pathway might play a role in avoiding the progression of early-stage disease [146].

SRC is a kinase located at cell–matrix adhesions that can be directly activated by the c-MET pathway, together with several ligands, such as EGF, HGF, platelet-derived growth factor (PDGF), VEGF and integrin and Eph receptor (EphA2) [147]. SRC participates in the modulation of integrin adhesions, cadherin-mediated cell-cell adhesions, metalloproteinase expression and other processes related to the tumor microenvironment [148].

Several studies have shown that some FGFR molecular alterations lead to the constitutive activation of SRC, which at the same time is regulated by EGFR-dependent mechanisms, SRC being a possible resistance pathway to FGFR. This knowledge has been exploited as a rational for new drugs such as dasatinib, which works as a broad-spectrum TKI and simultaneously co-targets FGFR and SRC, using this dual blockade to reduce cell viability in urothelial cancer cell lines [149].

### 6.4. Targeting Bruton’s Tyrosine-Kinase (BTK)

Bruton’s tyrosine-kinase (BTK) is a non-receptor intracellular kinase encoded by the BTK gene that plays a crucial role in the development of B lymphocytes, being required for signal transmission from the pre-B cell receptor formed after successful rearrangement of immunoglobulin heavy chains. BTK contains a PH domain that binds to phosphatidylinositol 3,4,5-trisphosphate (PIP3), an interaction that induces phospholipase C phosphorylation and hydrolyzes PIP2 into two second messengers (IP3 and DAG), which are able to modulate the downstream signaling activity of mature B cells [150].

As a member of TEC-family kinases, besides playing a key role in B lymphocyte signaling and activation, BTK is also present in other myeloid-system components and even epithelial and endothelial cells, where it is relevant in cytokine-mediated intracellular signaling. It has proved to regulate PI3K-dependent cellular activation pathways, thereby participating in the control of cell cycle and survival, and it seems to be involved in the immune system balance and tumor immune-escape mechanisms, both influenced by paracrine and cytokine-mediated regulation of tumor microenvironment [151].

BTK-targeted therapies such as ibrutinib interfere with the activation of B lymphocytes [152], whose protumorigenic activity has been associated with downregulation of INF-gamma release via secretion of IL-10 and enhancing cell survival by NFkB signaling, preventing the cytotoxic activity of CD8+ lymphocytes [153].

Ibrutinib, a BTK-targeted therapy, is useful in hematological malignancies, such as lymphocytic leukemia/small lymphocytic lymphoma, mantle cell lymphoma and marginal zone lymphoma [154,155]. Ibrutinib could have a promising role in the treatment of mUC. A phase 1B/2 study of ibrutinib daily and weekly paclitaxel in 29 patients as the second or third line of treatment showed 41% ORR. The median duration of response was 4.2 months, mPFS was 3.6 months and mOS was 14.7 months [156].

### 6.5. Targeting AXL

Axl is a kinase from TAM family (a subfamily of mammalian RTKs consisting of Axl, Tyro3/Sky and Mer) that is ubiquitously expressed in different types of cells, where it plays roles in cell adhesion, intracellular signaling and regulation of the immune system [157]. Its main ligand is growth arrest-specific gene 6 (Gas-6), whose interaction with Axl receptor results in an anti-apoptotic effect via increasing the activity of PI3K/Akt, MAP-k, JAK/STAT and NFkB signal transduction pathways [158].

As well as other RTKs, Axl has been implicated in the pathophysiology of many tumors, since its overexpression seems to correlate with increased invasiveness and poorer prognosis, indicating that Axl has a strong oncogenic potential [159]. Axl is overexpressed in several drug-resistant cancer cell lines, such as nilotinib-resistant myeloid leukemia cells [160], lapatinib-resistant HER-2 positive breast tumor cells [161] and cisplatin-resistant ovarian tumors [162], suggesting that Axl may be involved in the development of QT-resistance.

In BC, Axl seems to be directly upregulated by a molecule known as Fra-1, which is highly expressed in 80% of invasive bladder tumors (compared to 42% in superficial BC) [163]. In a study where immunohistochemistry was performed on 65 BC cancer specimens prior to radical cystectomy, Axl immunopositivity was associated with unsuspected lymph-node metastases and reduced disease-specific survival [164]. This information suggests that expression of Axl correlates with poor prognosis and may be a potential therapeutic target in advanced BC.

## 7. Discussion

Urothelial carcinoma is the most common histological subtype of BC, which is one of most prevalent malignancies worldwide. Platinum-based CT is the mainstay in mUC treatment. However, approximately 15% of patients are initially resistant to this therapy, and almost all patients progress eventually, but only 25–55% of patients will reach second line treatment. In addition, some patients are ineligible for this therapy due to different comorbidities. Therefore, the outcome of these patients is poor; the mOS for patients fit for cisplatin is only 12–14 months [162]. Even though different new strategies have emerged in the last few years, the increasing knowledge of the molecular alterations found in UC has driven the development of several drugs as new therapies—an urgently unmet need in BC considering the dismal prognosis. The main advance in recent years has been the irruption of the ICI, particularly PD1/PDL-1 inhibitors, in different contexts of the first- and second-line treatment and maintenance strategy, resulting in a small percentage of patients remaining alive for long periods of time [163]. Furthermore, enfortumab vedotin, a fully human monoclonal antibody against Nectin4 conjugated to monomethyl auristatin E, showed in the EV-201 trial, an ORR of 42% (95% CI: 33.6–51.6%), with 9% of complete responders and a mOS of about 12 months in heavily pre-treated patients, leading to accelerated approval by the FDA [164]. Despite this, the development of new treatment strategies is mandatory in this field. Tyrosine kinases have emerged in recent years as promising targets in urothelial cancer.

One of the most promising targets in mUC is FGFR, with erdafitinib being the first therapeutic target approved at the moment. The long-term outcomes from the phase 2 BLC2001 study confirm the efficacy results observed in the interim analysis [61,62]. In addition, their role in prior lines or earlier stages is being evaluated, along with new strategies, such as the combination with other drugs. Although data from phase 3 trials are still pending, FGFR inhibitors are already included in different international clinical guidelines. However, as previously exposed, data on response rates are not as impressive as other targeted agents, such as RET inhibitors in RET-fusion positive non-small-cell-lung cancer, for example [165]. In this sense, efforts are focused in adequately selecting patients from a molecular point of view and improving the knowledge in primary and acquired resistant mechanisms that may ultimately help to increase the antitumor response achieved with these drugs or to find the adequate partner to be combined with. Nowadays, the only FDA-approved assay for the qualitative detection of FGFR alterations susceptible for erdafitinib therapy is the QIAGEN therascreen^®^ FGFR RGQ RT-PCR kit. This diagnostic test is able to identify two-point mutations in exon 7 (p.R248C (c.742C>T); p.S249C (c.746C>G)), two-point mutations in exon 10 (p.G370C (c.1108G>T) and p.Y373C (c.1118A>G)) and two fusions (FGFR3:TACC3v1 and FGFR3:TACC3v3) in the FGFR3 gene. However, other trials including different FGFR inhibitors are using other assays, such as RNAscope™ ISH with rogaratinib or FoundationOne CDx^®^ with infigratinib trials. Therefore, for further development in precision medicine in urothelial carcinoma, improving molecular profiling and establish consensus for concordance between tests is undeniable. In addition, different strategies to inhibit FGFR are being analyzed, such as monoclonal antibodies and FGF traps, currently under early development clinical trials, and the role of FGFR inhibition in different settings of the disease, such as the non-muscle invasive context [166,167].

Although an international consensus concerning the subdivision of MIBC has been reached, the implications it may have in daily practice are still unknown. Due to the common oncogenic mechanism and mutations found, it is believed that luminal papillary subtype may response to FGFR targeted therapy, whilst stroma and basal subtypes are supposed to be responsive to EGFR targeted therapies. Moreover, this classification may also be used in the screening for basket clinical trials based on the molecular alterations. Nonetheless, this classification needs more validation in order to be implemented in routine practice.

The epidermal growth receptor family, mainly focused in HER2, showed modest results in clinical trials. A high rate of HER2 alterations was reported in BC, and the availability of anti-HER2 targeted therapies, and their efficacy in other solid tumors harboring alterations in HER2, encouraged the development of those drugs for BC. Nowadays novel therapies are being tested in this setting of metastatic patients, such as DS8201a in combination with nivolumab (NCT03523572), RC48-ADC for HER2-overexpressing patients (NCT03809013), RC48-ADC for HER2-negative patients (NCT04073602) and PRS-343 (bispecific antibody to HER2/41BB) (NCT03330561). Additionally, in mUC exists a consistent rational for targeting angiogenesis as a therapeutic strategy. However, monoclonal antibodies against VEGF or VEGF traps, such as bevacizumab or ramucirumab, though having initial promising activity, have not reached the main goals in the phase III trials. In this sense, multi-TKI targeting VEGFR, among others, has been evaluated in urothelial carcinoma with different results, cabozantinb and lenvatinib being the most promising and feasible to combine with ICI with potential synergistic activity. Other tyrosine kinases are showing interesting results, such as the BTK inhibitor ibrutinib, in combination with paclitaxel achieving an ORR of about 41% in mUC patients heavily pre-treated. New strategies such as the combination of BTK inhibitors with IT are granted.

Tyrosine kinases have demonstrated to play a key role in mUC treatment nowadays. In the near future, new combinations of tyrosine kinase inhibitors with IT, CT or targeted agents, will continue offering improvements in survival outcomes and quality of life for patients with mUC.

## 8. Conclusions

The development of novel inhibitors of tyrosine kinase targets are changing the therapeutic landscape of patients with mUC. Erdafitinib is the first FGFR inhibitor that has reached approval by regulatory agencies for patients with mUC who have been previously treated. Beyond erdafitinib and FGFR, novel drugs and targets are under research, as monotherapy or in combination, and are surely changing the natural history of the urothelial cancer disease.

## Figures and Tables

**Table 1:** Muscle-invasive bladder cancer (MIBC) consensus molecular classification.

	Differentiation	MIBC	Oncogenic Mechanisms	Mutations	Possible Targeted Therapies	Median OS (Years)
Luminal Papillary	Urothelial/ Luminal	24%	FGFR3–55%	FGFR3–40%	FGFR targeted therapies	4
			CDKN2A–33%	KDM6A–38%		
			PPARG			
	
Luminal non-specified		8%	PPARG–76%	ELF3–35%		1.8
Luminal unstable		15%	PPARG–89%	TP53–76%		2.9
			Erb-B2–39%	ERCC2–22%		
			E2F3/SOX4–76%			
Stroma rich	Urothelial/	35%	EGFR	TP53–61%	EGFR targeted therapies	1.2
	Squamous			RB1–25%	ICI	
Basal/Squamous	Squamous	35%	EGFR	TP53–61%	EGFR targeted therapies	1.2
				RB1–25%	ICI	
Neuroendocrine-like	Neuroendocrine	3%	TP53-, RB1-	TP53–94%		<1
				RB1–39%		

FGFR—fibroblast growth factor receptor. CDKN2A—cyclin-dependent kinase inhibitor 2A. KDM6A—lysine demethylase 6A. PPARG—perixosome proliferator activated receptor gamma. ELF3—ETS-related transcription factor. EGFR—Epidermal growth factor receptor. ICI—immune-checkpoint inhibitor. Adapted from Kamoun et al. Eur Urol. 2020.

**Table 2:** Clinical trials of FGFR inhibitors.

Study Design (NCT Identifier and Code)	Study Phase	Experimental Treatment	Population	Estimated *n*	Primary Endpoint	Estimated Study Completion Date
BLC2001 (NCT02365597)	Phase II	Erdafitinib	mUC with FGR3 mutation or FGFR2/3 fusion afterchemotherapy treatment	236	ORR	30 June 2022 (Recruiting)
NCT03390504	Phase III	Erdafitinib Pembrolizumab	mUC with FGFR alterations as second or third line of treatment	631	OS	5 November 2021 (Recruiting)
NORSE study (NCT03473743)	Phase I/II	Erdafitinib+cetrelimimab Erdafitinib+(cis/carbo)platin	mUC with selected FGFR alterations	160	DLT	17 March 2023 (Recruiting)
NCT04172675	Phase II	Erdafitinib	NMIBC with FGFR mutations or fusions and recurred after BCG therapy	280	RFS	10 June 2026 (Recruiting)
NCT01004224	Phase I	Infigratinib	Solid tumors with FGFR alterations	208	DLP	8 October 2018 (Completed)
NCT04197986	Phase III	Infigratinib	UC with FGFR3 alterations as adjuvant treatment	218	OS	31 January 2025 (Recruiting)
NCT01976741	Phase I	Rogaratinib	Several solid tumors without/with FGFR alterations	168	DLP	11 March 2019 (Completed)
FORT-1 (NCT03410693)	Phase II/III	Rogaratinib	mUC with FGFR1/3 after platinum-based chemotherapy	172	ORR	27 October 2020 (Completed)
FORT-2 (NCT03473756)	Phase Ib/II	Rogaratinib+atezolizumab	UC with FGFR alterations as first line of treatment	210	DLP	4 September 2024 (Recruiting)
FIGHT-201 (NCT02872714)	Phase II	Pemigatinib	mUC with FGFR alterations	263	ORR	31 March 2021 (Active, no recruiting)
FIGHT-205 (NCT04003610)	Phase II	Pemigatinib+atezolizumab Pemigatinib	mUC with FGFR3 alteration and not eligible to cisplatin	6	PFS	31 January 2026 (Recruiting)
NCT02052778	Phase I	TAS 120	Tumors with FGF/FGFR alterations	386	DLT	29 May 2021 (Active, not recruiting)
NCT01948297	Phase I	Debio 1347-101	Tumors with FGFR 1, 2, 3 alterations	77	DLT	26 June 2020 (Terminated)
BISCAY (NCT02546661)	Phase I	AZD4547 AZD4547+durvalumab	MIBC who progressed prior line of treatment	156	DLT	14 February 2022 (Active, not recruiting)
NCT04045613	Phase I/II	Derazantinib Atezolizumab Derazantinib ± atezolizumab	mUC with FGFR alterations	306	ORR	Recruiting (May 2022)
NCT00790426	Phase II	Dovitinib	UC	48	OS	April 2012 (Completed)
NCT01732107	Phase II	Dovitinib	NMIUC with FGFR3 alterations	13	ORR	6 March 2017 (Completed)

**Table 3:** Most common FGFR inhibitor-associated adverse events (AEs).

Drug	AEs Any Grade (%)	AEs Grade 3/4 (%)	Reference
Erdafitinib	Hyperphosphatemia (77%)	Hyponatremia (11%) Stomatitis (10%) Asthenia (7%) Nail dystrophy (6%) Hand-foot syndrome (5%)	[61]
	Stomatitis (58%)		
	Diarrhea (51%)		
	Dry mouth (46%)		
	Central serous retinopathy (27%)		
	Onycholysis (18%)		
Infigratinib	Hyperphosphatemia (46.3%)	Hyperlipasemia (10.4%) Fatigue (7.5%) Anemia (7.5%) Hand-foot syndrome (7.5%) Hypophosphatemia (7.5%)	[68]
	Increase in serum creatinine (41.8%)		
	Constipation (37.3%)		
	Fatigue (37.3%)		
	Anemia (35.8%)		
Rogaratinib	Hyperphosphatemia (60%)	Fatigue (9%) Anemia (6%) Urinary tract infection (8%) Hyperlipasemia (8%)	[71]
	Diarrhea (49%)		
	Decreased appetite (36%)		
	Fatigue (24%)		
	Nausea (28%)		
	Urinary tract infection (11%)		
Pemigatinib	Diarrhea (40%)	Urinary tract infection (7%) Fatigue (6%)	[74]
	Alopecia (32%)		
	Fatigue (29%)		
	Constipation (28%)		
	Dry mouth (28%)		
Debio-1347	Hyperphosphatemia (76%)	Hyperphosphatemia (21%) Anemia (12%) Dyspnea (5%) ALT increased (3%) Stomatitis (3%)	[76]
	Diarrhea (41%)		
	Nausea (40%)		
	Fatigue (40%)		
	Constipation (38%)		
	Decreased appetite (33%)		
	Nail changes (31%)		

## Abbreviations

AE	Adverse Events
ALK	Anaplastic Lymphoma Kinase
BC	Bladder Cancer
BCG	Bacillus Calmette–Guérin
BCRP	Bruton’s Tyrosine-Kinase
COX-2	Cyclooxygenase-2
CRS	Central Serous Retinophaty
CT	Chemotherapy
DFS	Disease-Free Survival
DLL	Delta-like canonical NOTCH ligand
DLT	Dose-limiting toxicity
EGF	Epidermal Growth Factor
EGFR	Epidermal Growth Factor Receptor
FDA	Food and Drug Administration
FGF	Fibroblast Growth Factor
FGFR	Fibroblast Growth Factor Receptor
FISH	Fluorescence In Situ Hybridization
HDI	Human Development Index
HGF	Hepatocyte Growth Factor
HIP	Hypoxia Inducible Factor
HR	Hazard Ratio
IHC	Immunohistochemistry
iNOS	Inducible Nitric Oxide Synthase
IT	Immunotherapy
ICI	Immune Checkpoint Inhibitors
JAG	Jagged Canonical NOTCH ligand
MAPK	Mitogen Activated Protein-Kinase
MIBC	Muscle-Invasive Bladder Cancer
miRNAs	micro-RNAs
mOS	Median Overall Survival
mPFS	Median Progression–Free Survival
mTOR	Mamalian Target of Rapamycin
mUC	Metastatic Urothelial Cancer
NGS	Next-Generation Sequencing
NMIBC	Non-Muscle-Invasive Bladder Cancer
ORR	Overall Response Rate
OS	Overall Survival
PCNA	Proliferating Cell Nuclear Antigen
PDGF	Platelet-Derived Growth Factor
PFS	Progression-Free Survival
PI3K	Phosphatidylinositol-3-kinase
PKC	Protein kinase C
PLC	Phosphatidylinositol-Specific Phospholipase C
PUMA	p53-Upregulated Modulator of Apoptosis
RFS	Recurrence-free survival
RON	Recepteur d’Origine Nantais
SCC	Squamous Cell Carcinoma
TCGA	The Cancer Genome Atlas
TSC	Tuberous Sclerosis Complex
TGF	Transforming Growth Factor
TMB	Tumor Mutation Burden
UUT-TCC	Upper urinary tract transitional cell carcinoma
VEGF	Vascular Endothelial Growth Factor
VEGFR	Vascular Endothelial Growth Factor Receptor
